# Dental Welfare Foundation

**Published:** 1921-09

**Authors:** W. Linford Smith

**Affiliations:** 645 Liberty Avenue Pittsburgh, U.S.A.


### Dental Welfare Foundation

**645 Liberty Avenue**
**PITTSBURGH, U. S. A.**

August 26, 1921.

TO DENTAL MANUFACTURERS AND DEALERS:

At the June meeting of the American Dental Trade Association held at Hot Springs, a paper was read on the subject of Popularizing Dentistry. This paper appears in the August issue of proofs and will be of intense interest to every man in the trade. Reprint of the paper will be cheerfully forwarded if the magazine containing it has been mislaid.

At the Milwaukee meeting of the National Dental Association, a paper was read embodying the same general idea, and I am very happy to say that following its presentation, the plan was approved by that organization, and permission has been granted to make public announcement of that fact.

The campaign will be conducted under the name of the Dental Welfare Foundation with headquarters at Pittsburgh.

Dr. Otto U. King, Secretary of the National Dental Association, has been appointed as a trustee to represent the Association in the councils of the Foundation.

The original trustees appointed by the American Dental Trade Association to put the plan in operation, were A. S. Carman, J. C. Forstbauer, G. F. Jones, Wm. C. Smith and W. Linford Smith, Chairman.

The co-operation of the dental profession and trade in the dental education of the public can not result otherwise than to the incalculable benefit of humanity.

Dealers and salesmen will be supplied with samples of the cards it is proposed to distribute, accompanied by advertising matter, manual for the use of salesmen, etc., in advance of the intensive eight weeks' drive which starts October 1st.

Manufacturers using full page space in dental magazines have been asked to donate such space for the use of the Foundation in the October and November issues of all dental publications, and from present indications, total circulation of the cards will run very much in excess of the million originally contemplated.

Dealers subscribing for names on their own account will be able to more consistently ask their customers to support this movement in view of which each dealer in the trade is urged to furnish a list of at least 1,000 names.

The total cost of mailing the cards twelve times during 1922 to a list of 1,000 names is $180, which is a very moderate contribution to the cause considering what others are doing.

The suggestion is offered in this connection that to secure the co-operation of employes, dealers, in making up their lists, should ask them to submit names of people living in the district where they reside, who, in their opinion, stand in need of dental education.

Dealers to whom this suggestion appeals will confer a very great favor upon the trustees by forwarding at once typewritten lists of names accompanied by check.

We are going to have a million or more stencils to cut and the job can't be done over night.

It is also hoped that employers even in advance of receiving further information as to details of the campaign, will use every means at their command to enthuse members of their sales organizations as to the big movement which has been started, and which, through the co-operation of organized dentistry and the activities of the individual salesman will most certainly be finished.

In the meantime, this is to be considered merely as an announcement of present progress, coupled with a request that every man in the trade be on his mark, get set and ready to GO when the starter says the word.

Yours for a million—and then some,

W. LINFORD SMITH, Chairman.

